# Wild Origins of Macadamia Domestication Identified Through Intraspecific Chloroplast Genome Sequencing

**DOI:** 10.3389/fpls.2019.00334

**Published:** 2019-03-21

**Authors:** Catherine J. Nock, Craig M. Hardner, Juan D. Montenegro, Ainnatul A. Ahmad Termizi, Satomi Hayashi, Julia Playford, David Edwards, Jacqueline Batley

**Affiliations:** ^1^Southern Cross Plant Science, Southern Cross University, Lismore, NSW, Australia; ^2^Queensland Alliance for Agriculture and Food Innovation, The University of Queensland, St Lucia, QLD, Australia; ^3^Australian Genome Research Facility, St Lucia, QLD, Australia; ^4^Centre for Tropical Crops and Biocommodities, Queensland University of Technology, Brisbane, QLD, Australia; ^5^Queensland Department of Environment and Science, Brisbane, QLD, Australia; ^6^School of Biological Sciences and Institute of Agriculture, The University of Western Australia, Crawley, WA, Australia

**Keywords:** *Macadamia integrifolia*, Proteaceae, chloroplast phylogenomics, crop domestication, phylogeography, bottleneck

## Abstract

Identifying the geographic origins of crops is important for the conservation and utilization of novel genetic variation. Even so, the origins of many food crops remain elusive. The tree nut crop macadamia has a remarkable domestication history, from subtropical rain forests in Australia through Hawaii to global cultivation all within the last century. The industry is based primarily on *Macadamia integrifolia* and *M. integrifolia–M. tetraphylla* hybrid cultivars with Hawaiian cultivars the main contributors to world production. Sequence data from the chloroplast genome assembled using a genome skimming strategy was used to determine population structure among remnant populations of the main progenitor species, *M. integrifolia*. Phylogenetic analysis of a 506 bp chloroplast SNP alignment from 64 wild and cultivated accessions identified phylogeographic structure and deep divergences between clades providing evidence for historical barriers to seed dispersal. High levels of variation were detected among wild accessions. Most Hawaiian cultivars, however, shared a single chlorotype that was also present at two wild sites at Mooloo and Mt Bauple from the northernmost distribution of the species in south-east Queensland. Our results provide evidence for a maternal genetic bottleneck during early macadamia domestication, and pinpoint the likely source of seed used to develop the Hawaiian cultivars. The extensive variability and structuring of *M. integrifolia* chloroplast genomic variation detected in this study suggests much unexploited genetic diversity is available for improvement of this recently domesticated crop.

## Introduction

Understanding the relationships between domesticated and wild germplasm is important to guide introduction of novel genetic diversity into selective breeding populations, and to prioritize conservation of novel wild germplasm that may be useful in the future (Brozynska et al., 2016; Chen et al., 2017; Luo et al., 2017; Zhang et al., 2017). Most major crops are derived from northern hemisphere Monocotyledon (monocot) and core Eudicotyledon (eudicot) species that were first domesticated 1000s of years ago (Miller and Gross, 2011). For these crops, there have generally been few domestication events and a small portion of the available genetic diversity in the wild progenitor species was selected (Wright et al., 2005; Doebley et al., 2006; Haudry et al., 2007; Meyer and Purugganan, 2013). A long history of selection, dispersal, hybridization and introgression, can lead to divergence between domesticated and wild source germplasm often obscuring the geographic origins of domestication (Burger et al., 2008; Fuller et al., 2011; Meyer et al., 2012). In addition, depending upon the intensity of anthropogenic activity, the original populations may be disturbed or lost (Fuller et al., 2011). In contrast, for more recently domesticated crops, there is the potential to identify specific source populations, although pinpointing the geographic origins of domestication requires a detailed knowledge of the population structure of the progenitor species (Schmutz et al., 2014).

Macadamia is unique in comparison to other horticultural tree crops. *Macadamia* (F. Muell, *2n = 28*) is a subtropical rain forest genus in the Proteaceae, an early-diverging eudicot family that had diversified in Australia by the Late Cretaceous (Mast et al., 2008; Sauquet et al., 2009; Nock et al., 2014; Carpenter et al., 2015). The four species in the genus are endemic to the lowland subtropical rain forest of eastern Australia and have a discontinuous distribution from south-east Queensland to north-east New South Wales (Powell et al., 2010, 2014). Macadamia is one of few international food crops derived from either the basal eudicots or the flora of Australia. Two species, *Macadamia integrifolia* and *M. tetraphylla*, produce an edible high-value oil rich kernel. Although it was likely a component of the diet of the indigenous peoples of Australia, to our knowledge, there is no recorded evidence of cultivation prior to European occupation of the natural habitat of the genus in the mid 19^th^ century (Gross, 1995; Costello et al., 2009; Hardner et al., 2009). The first European contact with the genus was reportedly in 1848 (Smith, 1956) and the first cultivated macadamia may be a tree planted in 1858 by Walter Hill in the Brisbane Botanical Gardens that is still alive today. Early botanists exported macadamia seed in the mid to late 19^th^ century while the first orchards in Australia were established from the late 19^th^ century most likely with germplasm from proximally located native forest (McConachie, 1980; Hardner et al., 2009).

The expansion of macadamia as a commercial crop initially occurred in Hawaii from the 1920s (Wagner-Wright, 1995; Hardner et al., 2009; Hardner, 2016). The favored species for commercial production, *M. integrifolia*, was initially introduced to Hawaii in two separate events in the late 19^th^ century. The first introduction was by W. H. Purvis sometime between 1881 and 1885, with trees planted near Kukuihaele on the Big Island. Subsequently, R. A. Jordan introduced macadamia into Hawaii in 1892 with trees from this second introduction grown in Honolulu on Oahu (Hardner, 2016). With recognition of the eating quality of the kernel, commercial seedling orchards were established throughout the Hawaiian Islands from the 1920s with seedlings trees reportedly derived directly from the 19^th^ century introductions (Shigeura and Ooka, 1984; Hardner, 2016). Following the development in Hawaii of reliable grafting techniques in the mid 1930s, seedling orchards were surveyed to identify elite trees that were subsequently clonally propagated. The performance of selected cultivars was evaluated prior to commercial release to the industry for the establishment of new plantations (Hardner et al., 2009).

The macadamia industry has undergone rapid global expansion in the last 50 years. Australia, South Africa, Kenya, and United States are currently the largest producers and the crop is also cultivated in China, southeast Asia, South America, Malawi, and New Zealand. Future growth in global production is predicted following recent extensions in planting, particularly in China and South Africa^1^. A few pure *M. tetraphylla* cultivars are grown commercially in South Africa (Peace et al., 2005). However, most industry cultivars are *M. integrifolia* or hybrids of *M. integrifolia* and *M. tetraphylla*. The *M. integrifolia* cultivars developed in Hawaii account for the majority of current world production and important founders of current breeding programs (Hardner, 2016). Knowledge of the extent and structure of genetic diversity is important for future genetic improvement, particularly in crops such as macadamia which is clonally propagated. *Macadamia* is adapted to subtropical rain forest habitat and recent genomic evidence points to an expansion of gene families involved in plant defense and pathogen recognition (Nock et al., 2016). A wide range of pests and diseases impact macadamia productivity and the identification of population structure and natural genetic variation is likely to be important in the development of resistant varieties.

The chloroplast is a plant organelle originating from an ancestral free-living cyanobacterium through endosymbiosis and performs a fundamental role in plant metabolism including photosynthesis (Gray and Doolittle, 1982; Timmis et al., 2004). The structure and gene content of chloroplast genomes are generally well-conserved among photosynthetic land plant species. They contain a large single copy (LSC) and small single copy (SSC) region separated by two inverted repeat (IR) sequences and range in size from 107 to 218 kb (Palmer, 1991). In contrast to the bi-parentally inherited nuclear genome, the chloroplast genome of most flowering plants is maternally inherited without recombination. Consequently, the chloroplast genome has been particularly useful for studying the maternal evolutionary history, or seedline, of angiosperms. Until relatively recently, intraspecific studies in particular were based on limited variation found in short PCR-amplifiable regions of the genome (Taberlet et al., 1991; Hamilton, 1999; Provan et al., 2001; Shaw et al., 2007). The development of next generation sequencing (NGS) technologies has led to a massive increase in the availability of shotgun sequence data for many plant species. This enables recovery of whole chloroplast genome sequences using a range of different techniques. These include (i) assembly of long-range PCR amplicons (Cronn et al., 2008; Whittall et al., 2010), (ii) ‘genome skimming’- shallow sequencing of total DNA that provides deep sequencing of high-copy chloroplast DNA, followed by assembly to a reference genome (Nock et al., 2011; Straub et al., 2012; Bock et al., 2014; Dodsworth, 2015) and (iii) *de novo* assembly with deeper NGS read coverage where no reference sequence is available (McPherson et al., 2013; Izan et al., 2017). Variation in the chloroplast genome has provided important insights into the domestication origins of crops including apple and citrus (Nikiforova et al., 2013; Carbonell-Caballero et al., 2015; Daniell et al., 2016). Recently, the complete chloroplast genome *M. integrifolia* cultivar HAES 741 ‘Mauka’ was sequenced (Nock et al., 2014) enabling comparative analysis of chloroplast variability assessed through a genome skimming strategy. In this study, intraspecific chloroplast sequence variation is used to investigate the population structure of remnant macadamia germplasm and applied to infer the origins of macadamia domestication. In contrast to other perennial tree crops species, this may be feasible due to the persistence of many wild populations and the short domestication history of macadamia.

## Materials and Methods

### Plant Material and DNA Extraction

**FIGURE 1:**
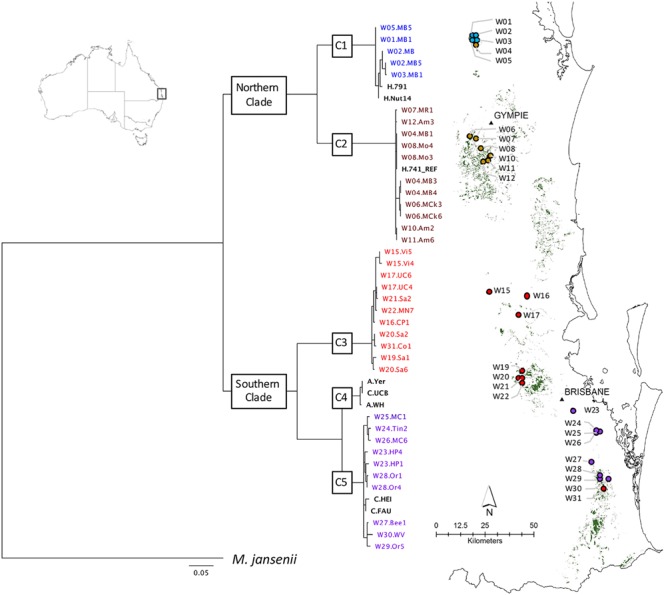
Geographic distribution of wild accessions relative to predicted remnant distribution (in green). Maximum likelihood phylogenetic tree (-lnL –32222.8) on left was inferred using RAxML from a 506 bp chloroplast SNP alignment of chloroplast haplotypes of 63 *Macadamia integrifolia* wild and domesticated accessions. The chlorotype of the reference genome (H.741_REF) was identical to those of three wild accessions (W08.Mo3, W08.Mo4, W04.MB1) and 18 other Hawaiian cultivated accessions. Bootstrap support for the two major clades and five sub-clades (C1–C5) was 100%. The outgroup was *M. jansenii*. Scale is substitutions per site. Cultivated accessions are in bold black and colors are used for the wild accessions and sites to identify the sub-clades to which they belong.

The National Macadamia Germplasm Conservation Program established *ex situ* plantings of clones of wild trees sampled in 1996 as cuttings from naturally occurring populations comprising most of the geographic range of the four species (Peace, 2002; Hardner et al., 2004). These *ex situ* plantings, located at Alstonville, Tiaro, and Burpengary in eastern Australia, were the source of most of the wild germplasm included in this study. The 64 accessions in total included (i) 37 samples from wild populations spanning the geographical distribution of *M. integrifolia* (Table 1A) (ii) cultivated germplasm including 26 *M. integrifolia* cultivars, selections, and cultivated trees (Table 1B) and (iii) a sample of *M. jansenii* as an outgroup in phylogenetic analyses. The wild accessions were originally sampled from 26 sites that were clustered into localities. A map of the predicted remnant distribution of macadamia was produced following habitat mapping methods outlined in Powell et al. (2010) to display the geographic location of the original sites sampled (Figure 1).

**Table 1:** Origin and source of wild **(A)** and cultivated **(B)** *Macadamia integrifolia* accessions.

(A) Wild accessions.						
Code	Germplasm origin	Sample origin	nReads	Coverage	Chlorotype	nSNPs
W01.MB1	Mount Bauple	CREEC Burpengary, QLD	144,435	204	C1.2	125
W02.MB3	Mount Bauple	CTH Alstonville, NSW	261,134	243	C1.1	124
W02.MB5	Mount Bauple	CTH Alstonville, NSW	180,247	169	C1.3	123
W03.MB1	Mount Bauple	CREEC Burpengary, QLD	111,165	155	C1.4	127
W04.MB1	Mount Bauple	CTH Alstonville, NSW	109,728	90	C2.1	0
W04.MB3	Mount Bauple	CTH Alstonville, NSW	43,003	101	C2.5	7
W04.MB4	Mount Bauple	CREEC Burpengary, QLD	141,397	197	C2.5	7
W05.MB5	Mount Bauple	CTH Alstonville, NSW	460,175	645	C1.2	121
W06.MCk3	Marys Creek	CTH Alstonville, NSW	390,181	363	C2.6	7
W06.MCk6	Marys Creek	CTH Alstonville, NSW	142,934	133	C2.6	7
W07.MR1	Mary River	CREEC Burpengary, QLD	40,774	57	C2.2	2
W08.Mo3	Mooloo	CTH Alstonville, NSW	75,256	175	C2.1	0
W08.Mo4	Mooloo	CTH, Alstonville, NSW	453,911	422	C2.1	0
W10.Am2	Amamoor Creek	CTH Alstonville, NSW	347,049	324	C2.4	2
W11.Am6	Amamoor Creek	CTH Alstonville, NSW	222,293	207	C2.4	2
W12.Am3	Amamoor Creek	CTH Alstonville, NSW	122,205	114	C2.3	2
W15.Vi4	Villeneuve	CTH Alstonville, NSW	375,635	351	C3.1	249
W15.Vi5	Villeneuve	CTH Alstonville, NSW	195,513	183	C3.2	251
W16.CP1	Campbells Pocket	MNGC Tiaro, QLD	47,966	112	C3.7	253
W17.UC4	Upper Caboolture	CTH Alstonville, NSW	263,225	245	C3.4	248
W17.UC6	Upper Caboolture	CTH Alstonville, NSW	1,160,658	1086	C3.3	249
W19.Sa1	Samford Valley	CREEC Burpengary, QLD	107,813	150	C3.10	247
W20.Sa2	Samford Valley	CTH Alstonville, NSW	23,356	55	C3.11	254
W21.Sa2	Samford Valley	CTH Alstonville, NSW	147,856	139	C3.5	249
W22.MN7	Mount Nebo	MNGC Tiaro, QLD	62,514	147	C3.6	254
W23.HP1	Holland Park	CTH Alstonville, NSW	115,706	108	C5.5	242
W23.HP4	Holland Park	CTH Alstonville, NSW	143,375	134	C5.4	243
W24.Tin2	Tingalpa	CTH Alstonville, NSW	225,728	210	C5.2	243
W25.MC1	Mount Cotton	CREEC Burpengary, QLD	46,977	65	C5.1	245
W26.MC6	Mount Cotton	CTH Alstonville, NSW	69,342	65	C5.3	246
W27.Bee1	Beenleigh	CREEC Burpengary, QLD	72,425	102	C5.10	243
W28.Or1	Ormeau	CTH Alstonville, NSW	177,786	165	C5.6	243
W28.Or4	Ormeau	CTH Alstonville, NSW	249,693	232	C5.7	241
W30.WV	Willowvale	Same as germplasm	263,981	370	C5.11	231
W29.Or5	Ormeau	CREEC Burpengary	173,190	243	C5.12	240
W31.Co1	Upper Coomera	MNGC Tiaro, QLD	41,874	98	C3.9	253
		Mean	195,729	214.4		157.5

*Germplasm origin refers to origin of the original tree (ortet) and sample origin refers to the location of the clone (ramet) from which samples were collected for this study. nReads, number of sequence reads mapped; Coverage, average read coverage; Chlorotype, sub-clade and chloroplast haplotype; nSNP, number of single nucleotide polymorphism differences relative to the reference genome, cultivar HAES 741; CREEC, Caboolture Region Environmental and Education Centre, Burpengary Australia; CTH, Centre for Tropical Horticulture, Alstonville; MNGC, Macadamia National Germplasm Collection.*

**Table 1:** **(B)** Cultivated germplasm.

Code	Germplasm origin	Sample origin	nReads	Coverage	Chlorotype	nSNPs
A.Yer	Yeronga, AU	Same as germplasm	30,066	70	C4.1	245
A.WH	Brisbane Botanical Gardens, AU	Same as germplasm	291,521	271	C4.2	235
H.246	HMNC Keauhou orchard, HI	CTH Alstonville, NSW	657,315	921	C2.1	0
H.294	HMNC Nutridge orchard, HI	CTH Alstonville, NSW	449,509	630	C2.1	0
H.333	HMNC Nutridge orchard, HI	CTH Alstonville, NSW	157,290	221	C2.1	0
H.344	HMNC Nutridge orchard, HI	CTH Alstonville, NSW	645,193	905	C2.1	0
H.425	HMNC Keauhou orchard, HI	UCR, CA	140,653	197	C2.1	0
H.508	HMNC Nutridge orchard, HI	USDA Waiakea, HI	53,892	76	C2.1	0
H.660	Deschwanden orchard, HI	CTH Alstonville, NSW	313,874	440	C2.1	0
H.741	Deschwanden orchard, HI	USDA Waiakea, HI	286,819	402	C2.1	0
H.HSp	HSC Kapulena orchard, HI	Honokaa, HI	406,422	570	C2.1	0
H.814	Malamka-Ki UH Exp. Station, HI	CTH Alstonville, NSW	482,659	676	C2.1	0
H.791	Bond Orchard, HI	CTH Alstonville, NSW	219,900	308	C1.5	122
H.Cwy	Coelho Way, Honolulu, HI	Same as germplasm	106,417	99	C2.1	0
H.Purv	Kapulena, HI	Same as germplasm	33,077	47	C2.1	0
H.TPN	Kapulena, HI	Same as germplasm	39,635	93	C2.1	0
H.TPS	Kapulena, HI	Same as germplasm	31,831	74	C2.1	0
H.Wai	Waipio Valley, HI	Same as germplasm	22,041	52	C2.1	0
H.Nut03	HMNC Nutridge orchard, HI	Same as germplasm	36,690	52	C2.1	0
H.Nut07	HMNC Nutridge orchard, HI	Same as germplasm	36,003	51	C2.1	0
H.Nut12	HMNC Nutridge orchard, HI	Same as germplasm	26,481	36	C2.1	0
H.Nut14	HMNC Nutridge orchard, HI	Same as germplasm	47,331	67	C1.6	126
H.Nut15	HMNC Nutridge orchard, HI	Same as germplasm	103,434	146	C2.1	0
C.UCB	UC Berkeley, CA	Same as germplasm	41,670	98	C4.1	247
C.Hei	Coronado, CA	Same as germplasm	13,254	31	C5.8	251
C.Fau	Faulkner Farm, CA	Same as germplasm	24,057	56	C5.9	248
		Mean	180,655	253.4		56.7

*Prefix of accession code indicates origin of germplasm. A, Australia; H, Hawaii; C, California; HMNC, Hawaiian Macadamia Nut Company; HSC, Honokaa Sugar Company; UH, University of Hawaii; UCR, University of California Riverside.*

With the exception of W30.WV, which was sampled from the original remnant tree, all wild accessions were sampled from the *ex situ* conservation plantings. The wild accession from Willowvale (W30.WV) is considered a maternal source of the Jordan introduction of *M. integrifolia* into Hawaii in the late 19^th^ century from which most of the initial seedling orchards are thought to have been derived (Hardner, 2016). Putatively planted and hybrid accessions suggested through collection notes and earlier molecular analyses (Peace, 2002) were not included in this study.

The 26 samples of cultivated germplasm included 11 Hawaiian cultivars: nine that were originally propagated directly from the early seedling orchards and are referred to using the Hawaiian Agricultural Experimental Station selection numbers (H.246, H.294, H.333, H.344, H.425, H.508, H.660, H.741, H.791), a cultivar selected by the Honokaa Sugar Company from their original seedling orchard (Honokaa Special, H.HSp), and an open pollinated selection derived from this cultivar (H.814). Other cultivated samples included three trees that are considered to be derived from the Purvis introduction (H.Purv, H.TPN, and H.TPS), a putative relative of the original Jordan introduction (H.Cwy), five samples from the Nutridge seedling orchard established in the 1920s near Honolulu (H.Nut03, H.Nut07, H.Nut12, H.Nut14, H.Nut15) from which five cultivars originated, and a seedling tree growing in the Waipio Valley believed to be from an old seedling orchard planted there in the 1930s (H.Wai) (Hardner, 2016). Three samples of old seedling trees from California were also included, a tree (C.UCB) planted in the 1879 the campus of the University of California, Berkley (Storey, 1977), a sample from a scion of an old macadamia cultivar Faulkner (C.Fau) that was selected from a planting at Santa Paula, CA that had been propagated from seed introduced from Florida about 1900 (Schroeder, 1954), and a tree (C.Hei) growing on the Coronado peninsula, San Diego that was planted about 1890 (Trask, 1962). Two samples were obtained from cultivated trees in Brisbane Australia, including the Walter Hill Tree planted in 1858 (A.WH) and a tree growing in the backyard of the suburb of Yeronga (A.Yer) planted approximately 60–70 years ago. A *M. jansenii* (C. L. Gross and P. H. Weston) individual sampled from the *ex situ* germplasm collection was included as an outgroup.

Fresh leaf material was collected, dehydrated using silica beads, and stored at room temperature prior to DNA extraction from single plants. Approximately 0.02 g of dried leaf tissue from each sample was ground in liquid nitrogen and total genomic DNA was extracted using a Plant DNeasy Mini Kit (Qiagen, Germany) according to manufacturer’s protocols. DNA concentration was quantified using a Qubit^®^ 2.0 Fluorometer dsDNA BR Assay system (Life Technologies, United States) with 2 μL of each DNA sample. The size and quality of the DNA extracts were also visualized on a 0.8% TAE agarose gel.

### Library Preparation and Sequencing

Genomic DNA was normalized to 50 ng/μl for library preparation. Sequence libraries for each sample were prepared using an Illumina Nextera XT DNA Library Preparation Kit following manufacturer’s instructions (Illumina, United States). Sequence libraries were quantified using a Bioanalyzer 2100 (Agilent, United States). Each *M. integrifolia* sample was barcoded with a unique index and libraries were pooled, and whole genome sequence data was generated using an Illumina HiSeq 2500 instrument at AGRF, Melbourne. Paired end sequence data (2 bp × 125 bp reads) were produced from pooled, indexed libraries of approximately 300 bp insert size. Sequence data (2 bp × 125 bp reads) for the unpooled, single *M. jansenii* library was generated using a MiSeq instrument at Southern Cross University with the library preparation procedures described above.

### Reference Mapping and SNP Calling

Quality control of raw sequence reads was performed using FastQC^2^, and adapter sequences and low quality bases were trimmed using Trimmomatic (Bolger et al., 2014). Reads ≥ 75 base pairs (bp) in length with a minimum Q-value of 20 were retained for further analysis. The complete chloroplast genome sequence of *M. integrifolia* cultivar 741 ‘Mauka’ (GenBank Accession No. KF862711) was used as a reference to identify SNP variants. Paired-end reads were mapped to the reference using SOAPaligner (Gu et al., 2013) allowing a maximum of two mismatches per read. Reads with low-quality alignments were identified and filtered out using SAMTOOLS with default parameters (Li et al., 2009). The programs Genome Analysis Toolkit, GATK (DePristo et al., 2011) and Picard Tools^3^ were used to optimize alignments by realigning reads around indels and removing duplicate reads following GATK best practices^4^. Following variant calling, the alignment was manually curated to remove low quality (Q < 10) sites. Mapping files (BAM) were used to identify SNPs for each sample in comparison to the reference genome of cultivar 741 using the SNP discovery pipeline SGSautoSNP that was developed for medium coverage resequencing data. In comparison, SAMtools/BCFtools requires extensive filtering to achieve similar true-positive rates of SNP discovery (Lorenc et al., 2012). Individual alignments were collated to produce a single variant call format (vcf) table for all samples using SAMTOOLS that was filtered to include only high-quality informative SNP sites with minimum coverage of 10x per sample. The program SnpEff (Cingolani et al., 2012) was used to annotate and predict the effects of SNPs in *M. integrifolia*.

### Phylogenetic Analysis

The vcf file with the final set of SNPs was converted into a concatenated sequence alignment of variable positions in fasta format using a custom perl script. Invariant positions were removed and a concatenated sequence alignment of variable positions was produced. *M. jansenii* was selected as outgroup because it is geographically isolated so does not naturally hybridize with *M. integrifolia*. The program JModeltest 2 was used to select an optimal substitution model for phylogenetic analysis (Darriba et al., 2012). Maximum likelihood analyses were conducted using Randomize Accelerated Maximum Likelihood RAxML 8.1.2 (Stamatakis, 2014) using raxmlGUI (Silvestro and Michalak, 2012) applying the most-likely substitution model (GTR+G, -lnL 3834.28, γ-shape parameter 99.81). To determine phylogeographic structure and the likely origin of cultivars and other cultivated germplasm, phylogenetic analysis was conducted independently on alignments of wild accessions and the total dataset including wild and cultivated accessions with gaps treated as missing data. In each case, to determine the optimal phylogeny and assess reliability, analyses implemented 1000 bootstrap replicates and 10 subsequent thorough maximum likelihood (ML) searches. Phylogenetic trees were viewed in FigTree 1.4.3. The relationships between distinct haplotypes were visualized using a statistical parsimony network (Templeton et al., 1992) constructed using TCS 1.21 (Clement et al., 2000).

To examine the relationship between SNP function and the phylogenetic structure, SNP variation was classified according to variation among and within phylogenetic clades and sub-clades. The predicted functional effect of a SNP was compared to its phylogenetic class, and a two-way chi-square analysis of the function-by-geographic structure contingency table of SNP characteristics was undertaken to test the hypothesis that the distribution of SNP function was independent of phylogenetic structure. For this test, sub-clade specific SNPs were collapsed into a single class and non-specific, intragenic, stop-gain, and stop-loss classes were excluded due to low numbers.

## Results

### Sequencing and Mapping

Raw sequence reads of 64 macadamia accessions were obtained and mapped to the chloroplast genome of *M. integrifolia* cultivar 741 (GenBank Accession No. KF862711). An average of 189,508 reads per *M. integrifolia* accession were mapped to the reference genome. Average read coverage was 214x for wild accessions and 253x for cultivated germplasm samples and ranged from 31 to 1086x per accession (Table 1). For the *M. jansenii* accession, 51,112,404 reads mapped to the reference with a mean coverage of 4,820x.

### Identification and Analysis of SNP Variation

**FIGURE 2:**
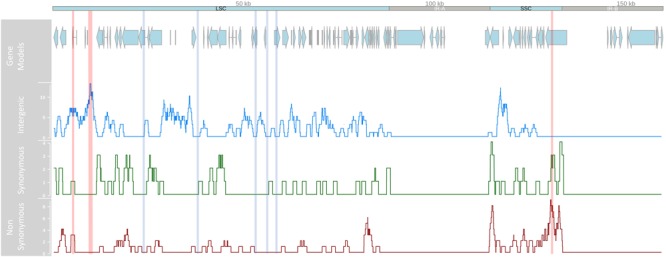
Linear representation of the *Macadamia integrifolia* chloroplast genome. Tracks from top to bottom show the position of protein-coding gene models in the large single copy (LSC), small single copy (SSC) and inverted repeat (IR-A, IR-B) regions, and location and number of intergenic, synonymous, and non-synonymous SNP/kb using a sliding 100 kb window. Vertical lines indicate regions of high (10–11 SNP/kb in red) and low (0 SNP/kb in blue) SNP density.

Following GATK mapping and manual curation, 506 non-redundant SNP sites were identified across the chloroplast genomes of 64 samples including the outgroup *M. jansenii*. Concurrent research indicates that the IR regions of the chloroplast genome are highly conserved between *Macadamia* species, for example only five IR single nucleotide polymorphisms (SNPs) were detected between *M. integrifolia* and *M. jansenii* (Nock, *unpublished data*). In this study, all intraspecific *M. integrifolia* SNPs were located in the LSC and SSC single copy regions only. Of these, 407 were variable within *M. integrifolia* and the average intraspecific single copy region SNP density was 3.8 SNPs per kb (Figure 2). Most variants were bi-allelic, however, 12 tri-allelic sites were identified including four within *M. integrifolia*. While the majority of intraspecific variants were located in the LSC region (310, 75.8%), SNP density was greatest in the SSC region (5.3 SNPs per kb, compared to 3.5 SNPs per kb in LSC). SNP variants were distributed across the single copy regions. However, SNP density was elevated in some regions with > 10 SNPs per kb spanning base positions 5–6 kb, 9–11 kb in the LSC and 130–131 kb in the SSC. Alternatively, some sections of the LSC were highly conserved with no SNPs detected within 23–24.5, 37–38.5, 52.5–54, 55.5–57, and 57.8–59.9 kb (Figure 2). Of the 407 intraspecific variant sites, 242 (59.5%) were located in non-coding regions and 165 (40.5%) were in exons. Variant sites within exons were located in 48 of 78 genes (61.5%) in the chloroplast single copy regions with most containing a single SNP. Thirteen genes were affected by > 3 variant positions, and the most variable genes were *ycf1* and *ndhF* with 30 and 17 SNP sites respectively (Table 2). Based on 506 non-redundant SNP sites, the non-synonymous to synonymous SNP ratio was 1:2. Three exonic variants were nonsense mutations, 97 were missense and 64 were silent. Among the non-synonymous mutations identified, nonsense mutations affected only two genes, *ndhF* (2) and *rpl16* (1), while missense mutations were detected in 36 genes (Supplementary Table S1).

**Table 2:** Highly variable *Macadamia integrifolia* chloroplast protein coding genes.

Gene	Protein	Length bp	N n	S n	SNP	Density SNP/kb
*ycf1*	Putative protein TIC214	5538	22	8	30	5.42
*ndhF*	NAD(P)H-quinone oxidoreductase subunit 5	2220	12	5	17	7.66
*rpoC2*	DNA-directed RNA polymerase subunit beta”	4185	6	5	11	2.63
*rpoB*	DNA-directed RNA polymerase subunit beta	3213	3	5	8	2.49
*matK*	Maturase K	1530	5	2	7	4.58
*psaA*	Photosystem I P700 chlorophyll a apoprotein A1	2253	2	5	7	3.11
*rpoA*	DNA-directed RNA polymerase subunit alpha	987	5	0	5	5.07
*ndhA*	NAD(P)H-quinone oxidoreductase subunit 1	2194	3	1	4	1.82
*rps16*	30S ribosomal protein S16	1090	3	1	4	3.67
*ndhD*	NAD(P)H-quinone oxidoreductase chain 4	1506	2	2	4	2.66
*psaB*	Photosystem I P700 chlorophyll a apoprotein A2	2205	2	2	4	1.81
*atpA*	ATP synthase subunit alpha	1524	1	3	4	2.62
*atpI*	ATP synthase subunit a	744	1	3	4	5.38

*Genes containing four or more SNPs, single nucleotide polymorphisms; bp, base pairs; N, non-synonomous; S, synonymous; kb, kilobase.*

#### Shared Haplotypes

**FIGURE 3:**
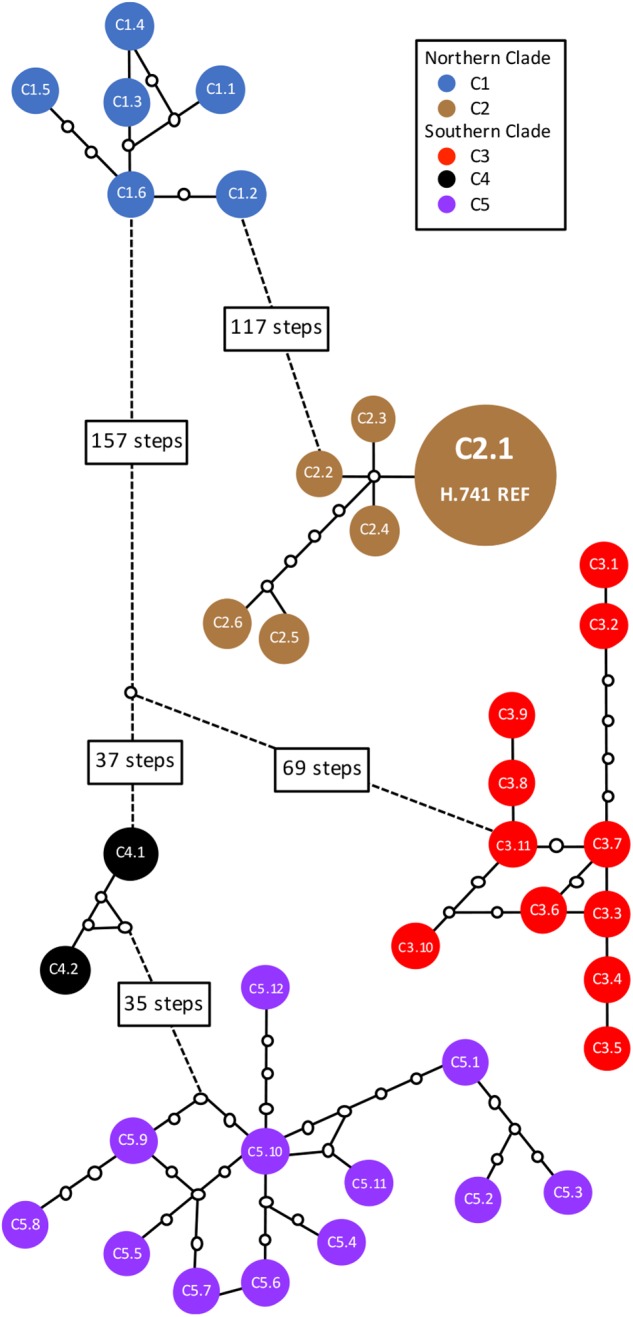
Statistical parsimony network of 38 distinct chlorotypes from 63 wild and cultivated accessions of *Macadamia integrifolia*. Solid colored circles represent chlorotypes; connecting lines are mutational pathways between haplotypes; white circles are extinct or unsampled haplotypes; longer pathways are represented by dotted lines with text boxes showing the number of mutational steps. The chlorotype for each sample is listed in Table 1. Chlorotype C2.1 of the reference genome (H.741 REF) was shared with three wild accessions (W04.MB1, W08.Mo3, W08.Mo4) and 18 other Hawaiian cultivated accessions.

In total, 38 distinct chloroplast haplotypes (chlorotypes) were identified, with one to 257 chloroplast SNP differences between them (Figure 3). The reference chlorotype of Hawaiian cultivar HAES 741 (H.741) was shared with two wild accessions sampled from the Mooloo site (W08.Mo3 and W08.Mo4) and one from a Mt Bauple site (W04.MB1). Chlorotypes were identical among samples from the same site (W04.MB3 and W04.MB4; W06.MCk3 and W06.MCk6), across sites within the Mt Bauple locality (W01.MB1 and W05.MB5) and within the Amamoor locality (W10.AM2 and W11.AM6). However, multiple chlorotypes were also found at other sites from which multiple accessions were sampled (W02, W04, W15, W17, W20, W23, and W28). The reference chlorotype was also identical to those of nine other Hawaiian cultivars (H.508, H.246, H.HSp, H.660, H.814, H.294, H.425, H.344, H.333) and nine other cultivated Hawaiian accessions (H.Nut03, H.Nut07, H.Nut12 and H.Nut15, H.Cwy, H.Purv, H.TPS, H.TPN, H.Wai). Interestingly, a single chlorotype was shared by two cultivated trees: one a backyard tree planted in Brisbane, Australia (A.Yer) and the other planted at the University of California Berkeley, United States (C.UCB). Of 21 Hawaiian accessions included in the study there were three distinct chlorotypes including the reference (cultivar 741), cultivar H.791 and H.Nut14. Cultivar H.791 and H.Nut14 differed from the reference chlorotype at 122 and 126 SNP positions respectively (Table 1).

### Phylogenetic Analysis

A concatenated sequence alignment of 506 SNP positions was used for phylogenetic analysis. The proportion of gaps was 1.41% and GC content was 63.4%. The best maximum likelihood tree (lnL = -32222.8) produced with the GTR+G model for all accessions shared the same topology as the best tree from analysis of a reduced dataset containing wild accessions only (lnL = -3221.4). Phylogenetic reconstruction revealed five well-supported clades. The tree was rooted with *M. jansenii* as the outgroup and there was maximum 100% bootstrap support for each of the two major clades and five sub-clades, C1–C5 (Figure 1).

#### Structure of Wild Populations

There was a clear relationship between the phylogenetic structure and geographic origin of the wild accessions of *M. integrifolia* in this study (Figure 1). Two major clades were identified. The northern clade contained all accessions sampled from sites around Amamoor in the Gympie region north to Mt Bauple which is the northern limit of *M. integrifolia.* The southern clade contained all wild accessions from sites from Villeneuve approximately 70 km northwest of Brisbane south to Upper Coomera approximately 50 km southeast of Brisbane. Within the northern clade, sub-clade C1 included accessions from four wild sites within the Mt Bauple region while sub-clade C2 included all accessions from Gympie to Amamoor. However, sub-clade C2 also included trees from a fifth Mt Bauple site (W04.MB). Accessions from wild sites to the south of the Amamoor region belonged to two sub-clades (C3 and C5) of the major southern clade. Sub-clade C3 contained accessions from sites between Amamoor and the Brisbane river, with the exception of a single accession from a site south of the Brisbane river (W31.Co1). Sub-clade 5 contained all other accessions from sites south of the Brisbane River from Holland park (W23.HP) to Willowvale (W30.WV).

Divergences within sub-clades were very shallow compared to the deeper divergences between the northern and southern clades, and sub-clades C1–C5 (Figure 1). Mutational steps separating the 13 northern and 25 southern haplotypes ranged from a minimum of 195 to 256, and within sub-clades from one to six to 16 steps (Figure 3). Further evidence of the phylogenetic structure of chloroplast variation in *M. integrifolia* was provided by the distribution of SNP variation among phylogenetic clades. Of 407 SNP variants in total, the majority (84.3%) were diagnostic for clades or sub-clades. There were 106 fixed differences between the northern and southern clades while 237 sub-clade specific SNPs were fixed (Table 3). A chi-square test for independence between clade-level phylogenetic structure and SNP annotation class (non-coding, synonymous, and missense) was not significant (*P* = 0.486). Most of the SNP variants were located in non-coding regions (244) or were synonymous mutations (64) and predicted to have no effect on gene function.

**Table 3:** Distribution of *Macadamia integrifolia* chloroplast SNP variation by annotation type and phylogenetic structure.

	SNP annotation type						
	Non-coding			Non-synonymous			
Phylogenetic class	Intergenic	Intragenic	Synonymous	Missense	Stop gain	Stop loss	Total
Clade specific	54	5	19	27	1		106
Sub-clade specific							
C1	28	3	10	8			49
C2	28	3	11	17			59
C3	33		10	13			56
C4	11		1	3			15
C4+C5	17	1	3	4			25
C5	19		5	9			33
Within sub-clade	16	1	4	12			33
Non-specific	23	1	1	4		2	31
**Total**	**229**	**14**	**64**	**97**	**1**	**2**	**407**

*Clade specific, polymorphisms fixed within the northern and southern clades. Sub-clade specific, fixed polymorphisms within each sub-clade (including C4+C5). Within sub-clade, variable within a single sub-clade only. Non-specific, unrelated to phylogenetic structure.*

#### Structure of Cultivated Accessions

The chlorotype of the reference accession (H.741), and the other 18 Hawaiian accessions included in this study were identical to the independently assembled reference genome of cultivar 741 (Nock et al., 2014), and were located within sub-clade C2 of the northern clade (Figure 1). The two Hawaiian accessions with unique chlorotypes different to the reference genome (H.791 and H.Nut14) belonged to the most northern sub-clade C1 that also included all accessions from four sites W01, W02, W03, and W05 at Mt. Bauple. None of the accessions from cultivated germplasm were associated with sub-clade C3. Two closely related Californian accessions (C.Fau and C.Hei) belonged to the southernmost sub-clade C5, and differed by a minimum of four substitutions from the wild accessions within this sub-clade. The southern sub-clade C4 was exceptional in that it contained only cultivated accessions including the Walter Hill tree (A. WH) and two other planted trees that shared identical chlorotypes (A.Yer and C.UCB).

## Discussion

This study demonstrates that phylogeographically structured intraspecific chloroplast sequence variation can be used to locate the wild sources of cultivated macadamia germplasm. For crops with long histories of domestication and multiple origins, their geographic origin often remains unresolved (Meyer et al., 2012). In contrast, commercial macadamia production developed only recently and wild populations may have been relatively undisturbed prior to the 19^th^ century. Global macadamia production is primarily based on grafted cultivars selected through breeding programs in Hawaii that were released less than 100 years ago, and may be only one to three generations from the wild (Hardner, 2016). The results of this study suggest that these cultivars were derived from a narrow seed pool and provide evidence for a genetic bottleneck in the maternal lineage of this recently domesticated nut crop.

### Phylogeographic Structure of *Macadamia integrifolia*

Phylogenetic analysis of chloroplast genomic variation revealed a latitudinal population structure of wild *M. integrifolia* germplasm, suggesting long-term regional isolation of maternal lineages (Figure 1). The deep divergence between northern and southern clades is indicative of an historical barrier to seed dispersal north of Brisbane, between the Brisbane and Mary River catchments. This finding is concordant with the only previous intraspecific genetic analysis of *M. integrifolia*, using nuclear randomly amplified DNA fingerprinting (Peace, 2002). The two major clades of *M. integrifolia* identified in this study are located within two separate subtropical refugia, or centers of endemism, defined by Weber et al. (2014). In addition, some of the suitable habitat in the region dividing the northern and southern clades is occupied by *M. ternifolia* (F.Muell) and hybrid populations (Costello et al., 2009; Hardner et al., 2009). These factors are likely contributors to the divergence between northern and southern *M. integrifolia* populations. Limited comparative chloroplast genome data for other Proteaceae taxa precludes reliable dating of intraspecific *M. integrifolia* divergences at this time. However, a crown age, for the subtropical genus *Macadamia* and its most recent common ancestor, of approximately 7 Mya was inferred from a fossil-calibrated phylogeny of the tribe Macadamieae taxa based on six chloroplast and nuclear genes (Mast et al., 2008). This period was coincident with the late Miocene contraction and fragmentation of rain forest habitat and aridification of much of the Australian continent (Byrne et al., 2008). During the Pliocene (approximately 5.3 to 1.8 mya) subtropical rain forest is thought to have persisted only on some regions of the Great Dividing Range and east coast with subsequent expansion and contraction during glacial and interglacial periods during the Quaternary (Byrne et al., 2011; Weber et al., 2014).

Further geographic structuring of genetic variation was detected within each of the major northern and southern clades. In the north, sites from Mt Bauple (C1) and Gympie (C2) regions formed two distinct clades. In the south, two clades including trees from sites north-west of Brisbane (C3) and south of Brisbane to the Gold Coast (C5) were separated by the Brisbane River Valley. Extensive evidence supports the existence of multiple biogeographic barriers in eastern Australia that led to vicariance events in rain forest restricted flora and fauna, including the speciation of *Macadamia* (reviewed in Weber et al., 2014; Bryant and Krosch, 2016). Spatial habitat modeling has been used to predict historical and remnant *M. integrifolia* habitat (Powell et al., 2010, 2014). Fragmentation and numerous gaps in the distribution of suitable habitat were identified particularly in the region separating the northern and southern clades, and the Brisbane River Valley separating the southern subclades (Figure 1). Our findings suggest that genetic divergence within *M. integrifolia* was the consequence of multiple barriers to seed dispersal between the lowland coastal ranges of subtropical eastern Australia. There was limited evidence for admixture between sites within the northern and southern regions, supporting the assumption that most of the sites sampled were remnant vegetation. Individuals from one Mount Bauple site (W04) were more closely related to those from the Gympie region than to those of proximally located sites suggesting that this site may contain translocated germplasm (Figure 3). Similarly, one tree from Upper Coomera (W31) on the Gold Coast belonged to a sub-clade (C3) that otherwise included only wild trees from sites north of Brisbane. The geographic-genetic discordance of individuals from these two presumed wild sites could be due to the long distance, possibly human-mediated, translocation of seed. Increased sampling and further research is needed to understand the extent of historical and more recent human-mediated dispersal in this species.

### Wild Origins of Macadamia Domestication

The high chloroplast variability and geographic structure of this variation support the use of chloroplast sequence data to identify wild origins of cultivated germplasm in macadamia. This study sampled three distinct maternal lineages, or chlorotypes, from the Hawaiian germplasm. All first generation cultivars that were selected from seedling orchards established from the early 1920s to the mid 1930s shared a single chlorotype. Subsequent Hawaiian cultivars and selections were from progeny of these selections (predominantly H.246) or from germplasm that was introduced into Hawaii in the 1950s (Hardner, 2016). This chlorotype was shared by all cultivars and cultivated germplasm sampled from Hawaii with the exception of H.791 and H.Nut14. The same chlorotype was also present in three wild trees from sites at Mooloo and Mt. Bauple suggesting that the maternal linage of almost all Hawaiian cultivars may trace back to one wild site, and perhaps even a single tree within a site. The Mt Bauple region and Mooloo valley, south west of Gympie in the north of the *M. integrifolia* distribution are still relatively undisturbed compared to other parts of the predicted pre-colonization distribution of macadamia. It is possible that the original trees from which seed was collected and taken to Hawaii may still be alive today. The differentiation of two other Hawaiian accessions (H.791 and H.Nut14) demonstrates some diversity in the maternal lineages of Hawaiian germplasm. Their likely ancestral origin is Mt Bauple given that their chlorotypes belong to a clade that otherwise includes only individuals from this region. Although closely related, the chlorotypes of H.791 and H.Nut14 differ by three mutational steps from each other so must have been derived from seed from different wild trees (Figure 3). These results agree with an earlier study suggesting that the Hawaiian cultivars originated from the north of the *M. integrifolia* distribution (Peace, 2002). Previous studies have examined genetic relationships among macadamia cultivars. Moderate variation was identified among Hawaiian cultivars using 16 allozyme loci although most alleles were shared (Aradhya et al., 1998). Subsequent analyses based on dominant AFLP and DNA RAF markers placed Hawaiian cultivars in separate but closely related *M. integrifolia* clusters (Steiger et al., 2003; Peace et al., 2005).

### Contribution of Reported Introductions to Hawaiian Domesticated Germplasm

Historical records on the development of the Hawaiian macadamia industry may provide some evidence about the contribution to the Hawaiian germplasm of different introductions and the wild origin of these introductions.

#### The Purvis Introduction

There is limited information on the possible origin of the first introduction of macadamia by W. H. Purvis into Hawaii. Hardner (2016) suggests that Purvis may have obtained germplasm in England in late 1882. He personally records taking Wardian cases of plants with him on his departure to return to Hawaii although the source of this material is unknown. Alternatively, seed (or plants) may have been obtained when he stopped in Sydney for a month on the same voyage. Some reports suggest that *M. ternifolia* was also included in the Purvis introduction. The samples H.TPN, H.TPS, and H.Purv are believed to represent the original Purvis plantings (Wagner-Wright, 1995; Hardner, 2016). The trees H.TPS and H.TPN grow in Kapulena, about 4.5 km from Purvis’ House in Kukuhaele. Honokaa Special (H.HSp) and H.Wai are reportedly from the Honokaa Sugar Company seedling orchard, while cultivar H.791 was reportedly selected from the Bond seedling orchard which was planted around 1922 with seedlings possibly supplied by Honokaa Seedling orchard. Samples included in this study that are presumed to represent the Purvis introduction all shared the same chlorotype with the exception of cultivar H.791.

#### The Jordan Introduction

The second documented introduction of macadamia to Hawaii was reportedly *M. integrifolia* seed shipped from Queensland in 1892 (Hamilton and Storey, 1956; Shigeura and Ooka, 1984). An old tree sampled in Hawaii (H.Cwy) is believed to be closely related to the Jordan introduction based on: (i) the age of the tree inferred from its large size, and (ii) the proximity, less than 80 m, to the last known survivor of the original six trees of the Jordan introduction (Shigeura and Ooka, 1984). Cultivars H.294, H.333, H.344, H.508 and other samples from the Nutridge orchard (H.Nut, Table 1B) are also thought to represent the Jordan introduction although this orchard may also have had minor contributions from other (*M. tetraphylla* and Purvis) sources. The Keauhou orchard, from which H.246 and H.425 were selected, was reportedly planted with germplasm that was only sourced from the Jordan introduction. In addition, Hardner (2016) suggests that the Deschwanden orchard, which produced H.660 and H.741, was established with germplasm collected from the Nutridge orchard. All 14 samples that are presumably representative of the Jordan introduction also shared the reference H.741 chlorotype with the exception of one (H.Nut14) from the Nutridge orchard.

Results from this study suggest that both the Jordan and Purvis introductions were derived from seed from at least three trees from northern *M. integrifolia* populations – the Jordan introduction from a lineage at Mooloo or Mt Bauple, and the Purvis introduction from a different Mt Bauple lineage. It has been suggested that a living tree at Willowvale in southeast Queensland (W30.WV) was the source of seed for the Jordan introduction (Lowndes, 1996). However, the chlorotype of this tree was most closely related to other samples in the most southern sub-clade C5 indicating that neither this tree, nor indeed any wild tree from this region, was a maternal parent of the Hawaiian germplasm included in this study. That the same maternal lineage is shared among the majority of Hawaiian samples in this study suggests that they were either derived from (i) the same introduction, or (ii) more than one introduction from the same region and the same maternal lineage. The other two maternal lineages identified include one cultivar (H.791) with *M. ternifolia* content (Peace et al., 2005).

### The Genetics of Extinct Wild Populations May Be Preserved in Cultivated Trees

Of interest is the sub-clade (C4) that includes two Australian and one Californian cultivated sample but included no wild accessions. This clade contains one of the oldest recorded cultivated macadamia planted by Walter Hill in the Brisbane Botanic Gardens, Brisbane (A.WH). Unfortunately, his records were destroyed in a flood so no information of the origin of this tree is available. This sub-clade also included a tree from a suburban backyard south of Brisbane (A.Yer) and another from the University of California (C.UCB). The distinctiveness of this sub-clade suggests that these trees may represent an extinct population, or a separate source of wild germplasm that was not sampled in this study. The phylogeographic structure of chloroplast variation suggests that these trees most likely trace back to a population south of the Brisbane River. Brisbane was settled in 1824 as a penal colony and much of the urban area was subsequently cleared due to development. It may be that wild populations existed in the region prior to the turn of the 20^th^ century when the population of Brisbane was small (135,000) compared to the present population of over two million. Although natural populations of macadamia have likely been lost since European occupation, our results suggest that planted trees in parks, gardens, and private backyards may represent a source of unique germplasm for future breeding.

The other two cultivated samples from California (C.Fau, C.Hei) trace back to wild sites associated with the southernmost sub-clade C5, and are most closely related to trees from sites south of Brisbane including Holland Park, Beenleigh and Ormeau. While their chlorotypes are closely related, there are no reports on the relationship between these trees.

### The Chloroplast Genome of *Macadamia integrifolia* Is Highly Variable

The chloroplast genome has been used extensively to resolve evolutionary relationships among plant species, and the capacity to detect variation has markedly improved since the advent of NGS (Chase et al., 1993; Parks et al., 2009; Moore et al., 2010; Soltis et al., 2011; Ruhfel et al., 2014). However, fewer studies have used whole chloroplast genome data to examine intraspecific diversity. Here we have sequenced the chloroplast genome of 64 accessions sampled from remnant wild and cultivated *Macadamia integrifolia* germplasm. Macadamia belongs to the Proteaceae, a large family of over 1700 species, spanning the remnant landmasses of Gondwana including Southern Africa, South America and New Zealand, and contains other species that are valued for food and floriculture. However, the first genomic data for the family became available only recently and we are unaware of any other phylogenomic study in the Proteaceae.

In total, 407 intraspecific polymorphisms and 38 distinct haplotypes were detected among 63 accessions with an average coverage of over 200x (Table 1). Chloroplast diversity in *M. integrifolia* is relatively high compared to that reported for other plant species including *Jacobaea vulgaris* (32 SNPs, 17 individuals), the model grass plant *Brachypodium distachyon* (298 SNPs, 32 haplotypes, 53 individuals), rapeseed *Brassica napus* (294 SNPs, 488 individuals) and Australian rain forest trees (6 to 240 SNPs per species, 12 individuals of 12 diverse species) (Doorduin et al., 2011; Van der Merwe et al., 2014; Qiao et al., 2016; Sancho et al., 2017). Relatively high genetic diversity is likely a consequence of the long-term persistence of genetically distinct populations in multiple stable subtropical rain forest refugia of eastern Australia through periods of historical climate variability (Weber et al., 2014; Rossetto et al., 2015). *M. integrifolia* is distributed over an approximately 250 km latitudinal range and is restricted to lowland subtropical rain forest on the coastal ranges of south east Queensland (Hardner et al., 2009; Powell et al., 2010, 2014). The large, hard-shelled seeds are thought to be dispersed by *Rattus rattus*, native rodents, gravity, and water (Pisanu, 2001; Neal et al., 2010; O’Connor et al., 2015). Despite an estimated 63% habitat loss and fragmentation due to land clearing, spatial habitat modeling indicates that a network of suitable *M. integrifolia* habitat persists (Powell et al., 2010).

Although the majority of variants were located in the LSC region of the chloroplast genome, SNP density was highest in the SSC region (5.26 SNPs per kb, compared to 3.52 in LSC). This finding has been reported for other plants including *Sesamum indicum* (Zhang et al., 2013) and *Panax ginseng* (Zhao et al., 2015) and is noteworthy because most universal primers and PCR-based intraspecific chloroplast studies assess variation only in the LSC. Regions of particularly high variability in *M. integrifolia* included the LSC intergenic spacer regions *trnQ-rps16* and *trnS-trnG-trnG*. Both have been identified as short, variable, and underutilized regions of the angiosperm chloroplast genome suitable for intraspecific phylogenetic studies (Daniell et al., 2006; Shaw et al., 2007). Within the SSC, the most variable regions included two genes, *ycf1* and *ndhF* (Table 2). *Ycf1* is the most rapidly evolving chloroplast gene (Dong et al., 2015) although its function across plant taxa remains unresolved (de Vries et al., 2015; Nakai, 2015; Bölter and Soll, 2017). The *ndhF* gene encodes a subunit of an NADH-specific dehydrogenase complex involved in photosynthetic electron transport (Yamori et al., 2016). Elevated sequence diversity and loss of function in chloroplast *ndhF* have been reported for a wide range of photosynthetic plant taxa (Wakasugi et al., 1994; Kim and Jansen, 1995; Kim and Chase, 2017). Our results are concordant with a recent phylogenetic analysis of 34 chloroplast genomes from *Citrus* and related species which found *ycf1, rpoC2, ndhF*, and *matK* to be the most variable chloroplast genes. There was evidence for positive selection of *ndhF* and *matK* exclusively in the Australian lineage (*Microcitrus* and *Eremocitrus*) suggesting that these genes may be involved in adaptation to contrasting climatic conditions (Carbonell-Caballero et al., 2015; Daniell et al., 2016).

## Conclusion

In this study, geographically structured variation of the *M. integrifolia* chloroplast genome was used to identify the wild origin and a maternal bottleneck in the Hawaiian cultivars that are the basis of the world macadamia industry. In addition, it appears that genetic diversity has been lost in the wild since European colonization, although some of this may be captured in cultivated trees. Comparison of chloroplast variation with that of the nuclear genome could test the hypothesis that the seed used to develop most of the Hawaiian cultivars was collected from a single tree, and will add greater insight into the genetics of the genus and crop.

## Data Availability

The datasets analyzed for this study can be found in the European Variation Archive EMBL-EBI (Project: PRJEB2832 Analyses: ERZ683764, https://www.ebi.ac.uk/ena/data/view/PRJEB28321).

## Author Contributions

CN prepared and drafted the manuscript, contributed to the study design, data analysis and interpretation and co-supervised AAT. CH established the *ex situ* conservation trials from which the wild germplasm used in this study was sampled, conceived and designed the study, collected Hawaiian, Californian and Australian cultivated samples, supervised AAT, and collaborated with CN to develop early versions of the manuscript. JM and DE managed data and performed bioinformatic analyses. AAT conducted initial laboratory work and analysis during a B.Sc. (Hons) research project. SH contributed to laboratory work, library preparation and sequencing. JP designed and supervised the original collection of wild germplasm from remnant populations. JB contributed to the data collection, analysis and interpretation and co-supervised AAT. All authors reviewed draft the manuscripts.

## Conflict of Interest Statement

The authors declare that the research was conducted in the absence of any commercial or financial relationships that could be construed as a potential conflict of interest.

## Footnotes

1 www.nutfruit.org/consumers/news/detail/inc-2017-2018-statistical-yearbook

2 https://www.bioinformatics.babraham.ac.uk/projects/fastqc/

3 http://broadinstitute.github.io/picard/

4 https://software.broadinstitute.org/gatk/best-practices/
