# Supplementary material for: Wild Origins of Macadamia Domestication Identified Through Intraspecific Chloroplast Genome Sequencing
Source: Front Plant Sci. 2019 Mar 21;10:334. doi: 10.3389/fpls.2019.00334 (PMC6438079; doi:10.3389/fpls.2019.00334)
Supplement: Supplementary file 1 [file Table_1.DOCX]

**Table S1.** Variable protein coding genes in the *Macadamia integrifolia* chloroplast genome. bp, base pairs. N, nonsynonomous. S, synonymous. nSNP, number of single nucleotide polymorphisms.

| Gene | Protein | Length  bp | N | S | nSNP | Density  SNP/kb |
| --- | --- | --- | --- | --- | --- | --- |
|  |  |  |  |  |  |  |
| ycf1 | Putative protein TIC214 | 5,538 | 22 | 8 | 30 | 5.42 |
| ndhF | NAD(P)H-quinone oxidoreductase subunit 5 | 2,220 | 12 | 5 | 17 | 7.66 |
| rpoC2 | DNA-directed RNA polymerase subunit beta’’ | 4,185 | 6 | 5 | 11 | 2.63 |
| rpoB | DNA-directed RNA polymerase subunit beta | 3,213 | 3 | 5 | 8 | 2.49 |
| matK | Maturase K | 1,530 | 5 | 2 | 7 | 4.58 |
| psaA | Photosystem I P700 chlorophyll a apoprotein A1 | 2,253 | 2 | 5 | 7 | 3.11 |
| rpoA | DNA-directed RNA polymerase subunit alpha | 987 | 5 | 0 | 5 | 5.07 |
| ndhA | NAD(P)H-quinone oxidoreductase subunit 1 | 2,194 | 3 | 1 | 4 | 1.82 |
| rps16 | 30S ribosomal protein S16 | 1,090 | 3 | 1 | 4 | 3.67 |
| ndhD | NAD(P)H-quinone oxidoreductase chain 4 | 1,506 | 2 | 2 | 4 | 2.66 |
| psaB | Photosystem I P700 chlorophyll a apoprotein A2 | 2,205 | 2 | 2 | 4 | 1.81 |
| atpA | ATP synthase subunit alpha | 1,524 | 1 | 3 | 4 | 2.62 |
| atpI | ATP synthase subunit a | 744 | 1 | 3 | 4 | 5.38 |
| rpl16 | 50S ribosomal protein L16 | 411 | 3 | 0 | 3 | 7.30 |
| ndhE | NAD(P)H-quinone oxidoreductase subunit 4L | 303 | 2 | 1 | 3 | 9.90 |
| ndhG | NAD(P)H-quinone oxidoreductase subunit 6 | 531 | 2 | 1 | 3 | 5.65 |
| petA | Cytochrome f | 963 | 2 | 1 | 3 | 3.12 |
| rpoC1 | DNA-directed RNA polymerase subunit beta’ | 2,776 | 2 | 1 | 3 | 1.08 |
| ndhH | NAD(P)H-quinone oxidoreductase subunit H | 1,182 | 2 | 0 | 2 | 1.69 |
| psbB | Photosystem II CP47 reaction center protein | 1,527 | 2 | 0 | 2 | 1.31 |
| rps11 | 30S ribosomal protein S11 | 393 | 2 | 0 | 2 | 5.09 |
| rps15 | 30S ribosomal protein S15 | 279 | 2 | 0 | 2 | 7.17 |
| rps2 | 30S ribosomal protein S2 | 711 | 2 | 0 | 2 | 2.81 |
| accD | Acetyl-CoA carboxylase transferase subunit beta | 1,503 | 1 | 1 | 2 | 1.33 |
| rpl32 | 50S ribosomal protein L32 | 174 | 1 | 1 | 2 | 11.49 |
| rpl33 | 50S ribosomal protein L33 | 210 | 1 | 1 | 2 | 9.52 |
| psbA | Photosystem II protein D1 | 1,062 | 0 | 2 | 2 | 1.88 |
| rps19 | 30S ribosomal protein S19 | 279 | 0 | 2 | 2 | 7.17 |
| ccsA | **Cytochrome c biogenesis** | 996 | 1 | 0 | 1 | 1.00 |
| cemA | Chloroplast envelope membrane protein | 690 | 1 | 0 | 1 | 1.45 |
| ndhJ | NAD(P)H-quinone oxidoreductase subunit J | 477 | 1 | 0 | 1 | 2.10 |
| psbD | Photosystem II D2 protein | 1,062 | 1 | 0 | 1 | 0.94 |
| psbZ | Photosystem II reaction center protein Z | 189 | 1 | 0 | 1 | 5.29 |
| rpl36 | 50S ribosomal protein L36 | 114 | 1 | 0 | 1 | 8.77 |
| rps12 | 30S ribosomal protein S12 | 140 | 1 | 0 | 1 | 7.14 |
| rps4 | 30S ribosomal protein S4 | 606 | 1 | 0 | 1 | 1.65 |
| ycf3 | Photosystem 1 assembly protein ycf3 | 1,971 | 1 | 0 | 1 | 0.51 |
| atpB | ATP synthase subunit beta | 1,497 | 0 | 1 | 1 | 0.67 |
| atpF | ATP synthase subunit b | 1,348 | 0 | 1 | 1 | 0.74 |
| atpH | ATP synthase subunit c | 246 | 0 | 1 | 1 | 4.07 |
| clpP | ATP-dependent Clp protease proteolytic subunit 1 | 2,036 | 0 | 1 | 1 | 0.49 |
| petB | Cytochrome b6 | 1,429 | 0 | 1 | 1 | 0.70 |
| petD | Cytochrome b6-f complex subunit 4 | 564 | 0 | 1 | 1 | 1.77 |
| petG | Cytochrome b6-f complex subunit 5 | 114 | 0 | 1 | 1 | 8.77 |
| psbC | Photosystem II CP43 reaction center protein | 1,422 | 0 | 1 | 1 | 0.70 |
| rpl14 | 50S ribosomal protein L14 | 369 | 0 | 1 | 1 | 2.71 |
| rps8 | 30S ribosomal protein S8 | 399 | 0 | 1 | 1 | 2.51 |
| ycf4 | Photosystem 1 assembly protein ycf4 | 555 | 0 | 1 | 1 | 1.80 |
|  |  |  |  |  |  |  |
|  | Total | 57,717 | 100 | 64 | 164 |  |
